# Medical Statistics

**Published:** 1836-01

**Authors:** 


### MEDICAL STATISTICS.

### *Historical and Statistical Account of la Maison Royale de Charenton,* by M. Esquirol.

M. Esquirol has given, in three successive numbers of the Annales d'Hygiene publique, an elaborate history of the large establishment for the insane at Charenton, two leagues to the east of Paris. The principal object which he has in view is to prove by statistical tables, that the portion of the establishment devoted to male patients requires to be rebuilt; and he has shown the necessity of such an alteration from the diminished mortality and increased proportion of cures among the females, whose apartments have been more recently and commodiously erected. This is an important subject, and of much local interest, but our space will not allow us to follow M. Esquirol in the history and description of the institution, which relate particularly to the subject he is interested in. The last article is of general importance, as it contains the results of M. Esquirol's practice during the eight years he has acted as principal physician at Charenton, and to this we shall confine ourselves, after introducing a few necessary preliminary details.

Insane patients of every age, sex, or rank, whatever may have been the cause, character, or duration of the disease, are admitted. The whole is under the direction of government. Fourteen beds are allotted to the poor of the canton, conformably to the original intention of the institution, which was founded for this laudable purpose by the brothers of the religious order of la Charité, about the year 1602. Sixty-five beds are also placed at the disposal of the government, who may also admit thirty-eight patients at a greatly diminished rate. These are usually given to naval and military officers, literary men, artists, &c. to whom their country is under some obligations, and who are not fit objects for the large pauper lunatic asylums of Paris. The remaining patients pay a certain annual sum. The whole are divided into three classes.

| | | | |
|---|---|---|---|
| The 1st class pays | | 1,300 | francs and upwards |
| 2d | | 1,000 | — |
| 3d | | 720 | — |

The regulations for the medical and general management of the patients, and of the institution, are well worthy of attention. There is one regulation which is peculiarly valuable: a bulletin of the state of each patient is sent every fortnight to the relations. The following is the formula:

MAISON ROYALE DE CHARENTON.
*Medical Bulletin.*

Mental Condition }

Bodily Condition }

Prognosis }

*Charenton, the* 183
PHYSICIAN IN CHIEF.

M. Esquirol replaced M. Royer-Collard as head physician in 1826, under whose care the institution had flourished in every respect. Since that date, M. Esquirol has kept accurate statistical tables of various particulars, and he is fully convinced, by thirty years' experience, of the utility of the information thus furnished.

The following table shows that the average number of admissions during the last eight years has been rather more than 194.

*Admissions.*

| Years | 1826 | 1827 | 1828 | 1829 | 1830 | 1831 | 1832 | 1833 | Total |
|---|---|---|---|---|---|---|---|---|---|
| Men . | 121 | 123 | 122 | 121 | 112 | 109 | 118 | 106 | 932 |
| Women | 89 | 82 | 82 | 71 | 74 | 82 | 79 | 66 | 625 |
| Total | 210 | 205 | 204 | 192 | 186 | 191 | 197 | 172 | 1557 |

Several local causes may tend to account for the diminution in the numbers during the last four years; such as the circumstances which since 1830 have kept many persons away from Paris, who formerly resided there; the increase and amelioration of provincial asylums; and the recent permission of those who are not indigent to enter the Bicetre and la Salpetrière, formerly exclusively devoted to paupers.

The next table gives the months during which the admissions took place, from which it appears, that during

| | | | | |
|---|---|---|---|---|
| Spring | (three months) there were | 406 | } 1,557. |
| Summer | do. | — | 445 | |
| Autumn | do. | — | 365 | |
| Winter | do. | — | 341 | |

The admissions were most numerous in July, and in the three summer months, and least so in September, and in the winter quarter: so that the number of the insane began to increase in Spring, arrived at its greatest amount in summer, diminished in autumn, and still more so in winter. There were also fewer men in proportion admitted in winter, and fewer women in spring.

*Table of Admissions relative to Ages.*

| AGES. | Sex. | | | AGES. | Sex. | | |
|---|---|---|---|---|---|---|---|
| To 20 years old | M | 82 | | From 55 to 60 | M | 37 | |
| | W | | 42 | | W | | 36 |
| From 20 to 25 | M | 119 | | From 60 to 65 | M | 35 | |
| | W | | 55 | | W | | 22 |
| From 25 to 30 | M | 135 | | From 65 to 70 | M | 25 | |
| | W | | 72 | | W | | 10 |
| From 30 to 35 | M | 130 | | From 70 to 75 | M | 4 | |
| | W | | 77 | | W | | 2 |
| From 35 to 40 | M | 106 | | From 75 to 80 | M | 2 | |
| | W | | 102 | | W | | 4 |
| From 40 to 45 | M | 105 | | From 80 to 85 | M | 1 | |
| | W | | 90 | | W | | 1 |
| From 45 to 50 | M | 83 | | From 85 to 90 | M | 0 | |
| | W | | 65 | | | | |
| From 50 to 55 | M | 68 | | | | 932 | 624 |
| | W | | 46 | | Total | 1556. | |

*Table of Admissions relative to Ages and Sexes, classed according to their frequency.*

| Men. | | Women. | |
|---|---|---|---|
| From 25 to 30 years | 135 | From 35 to 40 years | 102 |
| „ 30 to 35 ... | 130 | „ 40 to 45 ... | 90 |
| „ 20 to 25 ... | 119 | „ 30 to 35 ... | 77 |
| „ 35 to 40 ... | 106 | „ 25 to 30 ... | 72 |
| „ 40 to 45 ... | 105 | „ 45 to 50 ... | 65 |
| „ 45 to 50 ... | 83 | „ 20 to 25 ... | 55 |
| Before 20 years | 82 | „ 50 to 55 ... | 46 |
| „ 50 to 55 ... | 68 | Before 20 years | 42 |
| „ 55 to 60 ... | 37 | From 55 to 60 ... | 36 |
| „ 60 to 65 ... | 35 | „ 60 to 65 ... | 22 |
| „ 65 to 70 ... | 25 | „ 65 to 70 ... | 10 |
| „ 70 to 75 ... | 4 | „ 75 to 80 ... | 4 |
| „ 75 to 80 ... | 2 | „ 70 to 75 ... | 2 |
| „ 80 to 85 ... | 1 | „ 80 to 85 ... | 1 |
| „ 85 to 90 ... | 0 | “ 85 to 90 ... | 1 |
| | 932 | | 625 |

A comparison of both these tables shows, 1st. That the maximum of admissions takes place between 30 and 35 years. 2d. That from 20 to 35 the admissions, reckoned every five years, are raised to the same number, and have been stationary. 3d. That the admissions of men are more numerous from 25 to 30, and those of women from 35 to 40: next in frequency are the admissions from 30 to 35 for men, and from 40 to 45 for women. The male admissions from 20 to 25 hold the third rank in frequency, whilst the same age among women holds the sixth rank; from which M. Esquirol concludes (as he has done in his celebrated article on Insanity) that madness attacks men earlier than women. From 50 to 55, madness is rather less frequent. After 55 the admissions decrease rapidly in both sexes, but they are rather more numerous in women. These results are true as regards the absolute number of admissions; but on comparing them with the whole population of each age, it is proved that with the progress of years, the intellectual faculties become weakened and extinguished. More men are received at Charenton than women, owing to military and naval officers and others being gratuitously admitted by the government. From other researches of M. Esquirol it appears, that the proportion of males to females in 76,000 insane persons, is as 37 to 38, but that this difference varies with the climate, population, and manners in the same country.

*Table of Admissions relatively to the Civil Condition.*

| | M. | W. |
|---|---|---|
| Unmarried ... | 505 | 193 |
| Married ... | 387 | 363 |
| Widowers ... | 40 | |
| Widows ... | | 69 |
| Total of Men | 932 | |
| Total of Women | | 625 |
| General total 1557. | | |

It follows from this table, 1st. That the unmarried insane at Charenton are in proportion to the married, as 1 to 2,22; and that the unmarried men are to the women as 5 to 2. 2d. That the married insane, are to the admissions as 1 to 2; that there is very little difference in number between the men and women. 3d. That widows and widowers are but one fifteenth of the whole, and that widowers are to widows as 4 to 7. Bachelors are more frequently insane than married women; for insanity attacks men between 25 and 30, when men are the most under the dominion of their passions, and hardly think of marriages; whilst the women are generally married. The number of married females at Charenton equals that of married men.

Do the physical and moral sufferings of women after marriage expose them more frequently to insanity?

The next table refers to the professions and occupations of the insane at Charenton, which we shall not transcribe, as nothing more can be deduced from it than that no circumstances place an individual above the reach of this disease. During 1826, 1830, and 1831, the admission of annuitants (rentiers) a numerous class in Paris, was greater than during the five other years. This is accounted for by the "remboursement des rentes" (paying off of certain funds,) in 1825, and by the political events in 1830-31.

*Table of Admissions according to the Causes of Insanity.*

| | From 1826 to 1833. | | From 1826 to 1833. |
|---|---|---|---|
| Hereditary ... | 337 | Cholera .. ... | 3 |
| Onanism ... ... | 52 | Domestic grief ... | 278 |
| Licentiousness ... | 116 | Excess in study ... | 16 |
| Abuse of Mercury ... | 41 | Reverse of fortune ... | 49 |
| —— of wine and spirits | 134 | Gaming . ... | 5 |
| Insolation .. ... | 12 | Jealousy ... ... | 18 |
| Blows on the head ... | 20 | Love opposed ... | 37 |
| Suppression of habitual evacuations ... | 54 | Wounded self-love ... | 16 |
| | | Alarm ... ... | 35 |
| —— of habitual suppuration ... | 3 | Exalted devotion ... | 24 |
| | | Excess of joy ... | 2 |
| After delivery ... | 28 | Reading novels ... | 13 |
| Cerebral diseases ... | 17 | Political events ... | 32 |

The study of the causes of insanity is as difficult as it is important. The patients are unable to afford any information, and the relations are often either incapable of judging correctly, or are willing to deceive: some are sent without any information of their previous condition. This table is consequently far from perfect, but the results furnished by it are not without interest. It should be remembered that several causes, both exciting and predisposing, physical and moral, may act simultaneously. Of all diseases, insanity is most eminently hereditary. Of 1375 cases, 337 were ascertained to be hereditary, and M. Esquirol is convinced that this number is much under the mark. Errors in diet, and excess of every kind, either by gradually weakening the organs of sensibility, or briskly perverting their functions, frequently produce madness. Epilepsy, and particularly epileptic vertigo, always modifies the nervous functions of those so affected. Epileptics are highly susceptible and irritable; their fits terminate sometimes by furious mania, rarely by monomania, often by the most stupid dementia; but this consecutive disease is not of long duration, it generally goes off after some hours, or days, to be again brought on by a fresh fit; but when epilepsy has lasted many years, and the attacks have been more frequent, (particularly the vertigo,) the intellect becomes changed, weakened, and lost. M. Esquirol has found that short attacks of vertigo destroy the intellect more rapidly than complete attacks of epilepsy. Insanity has followed delivery 28 times, that is, in the proportion of 36 to 12 on the whole number of women admitted during eight years. This cause was more frequent in the Salpêtrière, amounting to one twelfth, which can be explained by the greater misery and destitution of the pauper females.

A greater number of physical than of moral causes were noted, which is contrary to the observations of both Pinel and Esquirol at the Salpêtrière: there, however, women alone were received, and females are more under the influence of moral emotions than men. It is also more difficult to ascertain moral than physical causes. Under the head domestic grief is included all those moral feelings which are called into action in the family circle, such as bad humour, discouragement in the domestic economy, cares for children, the loss of near relatives, professional jealousies, deranged circumstances; so that the large figure attached to this head, will cause no surprise. Alarm produced more instances of madness in 1830, than in the four preceding years. Before 1830, political causes were not remarked, but in that year there were thirteen cases, and in the next year fifteen cases, attributable to political changes. By comparing the frequency of madness in 1830-31 in certain professions, with these moral causes, it is evident that the social disturbances of this period exercised their influence on the production of insanity, not only by the alarm, but the reverses of fortune they occasioned in so many instances. This conclusion confirms one, at which M. Esquirol arrived in 1805, that the dominant ideas of every age, the state of society, and political commotions, considerably influence both the character and frequency of insanity. "I could sketch (says M. Esquirol) the history of my country from 1789 to the present day, from observing several maniacs, whose insanity was caused or characterized by some remarkable political event which took place in this period; and if I had to render an account of the greater number of suicides in 1834, and of the causes of their frequency, it would be sufficient for me to give an accurate history of the intellectual and moral state of society in France. We should see that the disease is old, but that new circumstances have aggravated it."

*Table of Admissions according to the Varieties of Insanity.*

| | M. | W. |
|---|---|---|
| Monomania | 372 | 343 |
| Mania | 334 | 211 |
| Dementia | 219 | 62 |
| Idiocy | 8 | 7 |
| Delirium alone | | 1 |
| | 933 | 624 |
| | Total 1557. | |

Monomania is the most frequent variety. It is in proportion to the whole admissions as 1 to 2,17; it affects women more frequently than men, relatively to the number of admissions of the two sexes: women are more frequently victims of sad and depressing emotions, and more exposed to melancholy with delirium. Mania, more frequent among men, was, relatively to the admissions, as 1 to 2,85: dementia as 1 to 5,54, and the proportion of men subject to it *much* greater than that of women. Idiocy was noticed 15 times only in 1,557 cases: it is rare in a civilized country like France, but not so in other countries; thus according to the statistics of Dr. Holst it abounds in Norway. Dr. Halliday shows that there are many idiots in Scotland; Sir G. Staunton has seen many idiots on the frontiers of Chinese Tartary: and what are the Cretins of Switzerland, of the Alps and Pyrenees, but idiots of the mountains? If Insanity is frequent, and idiocy rare in France, it is because the two are very different. Insanity is in the direct ratio of civilization; it is the product of intellectual and moral influences: idiocy, on the contrary, depends on the soil and on material causes. The causes of idiocy oppose the development of the organs, and intellect cannot manifest itself. In insane persons, the organs are well developed, but being over excited, reason is overthrown. It is so true that material influences are the cause of idiocy, that wherever civilization has penetrated and has modified these influences, the Cretins have diminished in number. Rainon has stated this of the Cretins of the Pyrenees; others have observed it in Switzerland, and any one may verify it by visiting those mountainous countries where civilization has increased the means of existence, and changed the mode of life of the inhabitants.

Three tables follow, one of the annual number of cures and deaths; another of cures relatively to sexes and seasons; and the third of deaths relatively to sexes and seasons: but as the general summary contains the really important matter, we shall omit the details on which it is founded. In eight years there were 518 cures; 514 returned to their relations without being cured; and there were 546 deaths. The total number of admissions was 1557, and the proportion of cures was as one to three. If from the total number of admissions, are subtracted 274 paralytics,

62 epileptics, and 15 idiots, in all 352 cases, which all practitioners regard as incurable, 1,205 will remain, and the cures being 516, the proportion is as 1 to 2,33. The proportion of cures might be still farther increased by deducting several cases of patients leaving when under improvement; or dying of other diseases when convalescent, &c. and by subtracting the large number of 492 patients, which were left in the hospital by M. Esquirol's predecessor uncured, the greater part of whom had lived there many years.

The cures of females, relatively to admissions, are greater in number than those of men. This M. Esquirol attributes to the improved state of that portion of the building devoted to females. The cures have been most numerous in October, and fewest in February; the men have been cured more often in November and July, the women in October and May. If the year is divided into four seasons of three months each, it will appear that autumn is most favourable to cures, and winter least so. The cures are more numerous in spring, increase still in summer, and attain their maximum in autumn. Thus

| | | |
|---|---|---|
| Winter three months | 92 cures | 518. |
| Spring „ | 123 | |
| Summer „ | 145 | |
| Autumn „ | 158 | |

Monomania, relatively to the number of admissions, is cured more frequently in women; mania on the contrary, is more often cured in men; for 160 men have been cured, and only 103 women. Dementia is hardly ever cured; and idiocy never, as it depends on defective organization.

*Cures relatively to the Forms of Insanity.*

| | Men. | Women. | Total. |
|---|---|---|---|
| Monomania | 123 | 128 | 251 |
| Mania | 160 | 103 | 263 |
| Dementia | 1 | 3 | 4 |
| | 284 | 234 | 518 |

The deaths have been, relatively to the whole number, as 1 to 3,75, that is, nearly one fourth. The proportion of deaths was greater among the men, 406 men having died, and 140 women; the proportion being as 1 to 29. The state of the building explains this. The mortality was at its maximum in winter, diminishing in spring and autumn, and arriving at its minimum in summer. Thus

| | | |
|---|---|---|
| Winter three months | 160 deaths | 546. |
| Spring „ | 139 „ | |
| Summer „ | 119 „ | |
| Autumn „ | 128 „ | |

Many circumstances, besides the improper construction of the portion of the building which contains the men, explain this great mortality. Recent cases are rarely sent to Charenton, and as has been previously mentioned, upwards of a fourth were paralytic, epileptic, or idiodic; in fact, incurable.

The examinations of all the patients after death were conducted with the most scrupulous attention, and a written statement of the appearances was added to the history of the case. Lesions of the brain and its membranes were found more frequently than diseases of the thoracic or abdominal viscera. The contrary was the case at la Salpêtrière, the field of M. Esquirol's previous practice; very few of the women in this latter establishment being paralytic. Indeed, the examinations of the bodies at both places have not explained the material conditions on which the delirium of insanity depends. New efforts may perhaps disclose the cerebral lesions which produce insanity, and our present ignorance should not discourage our attempts.

*Annales d'Hygiène Publique, Janvier*, 1835.

### II. *Official Report of the State of Lunatics in Norway in the Year* 1825. By Frederick Holst, m.d.

[With the view of rendering more complete our present illustrations of the Statistics of Insanity, we have transcribed the following tables from the very interesting work of Professor Holst, of Christiania, referred to in the preceding article.* Although published now seven or eight years, we have reason to believe that this document is scarcely known in this country: it has been recently referred to, with just commendation, by Dr. Prichard, in his admirable work on Insanity.—The data from which the following tables were drawn up, were procured from reports from the pastors of the several parishes in the kingdom of Norway, who had blank forms sent them for the purpose. They were to give in the name, age, and sex of each lunatic, with the nature of the disease, (whether mania, melancholia, dementia, or idiotismus), to assist them in which task the form contained clear descriptions of the four kinds: they were also to add its duration, probable cause, whether the patient was then, or ever had been subject to epilepsy, and whether he had had medical aid, and if so with what result. From these data the commissioners determined the prognosis, or division into curable and incurable cases, according to the rules laid down by Pinel, Esquirol, Georget, Heinroth, Cox, Hallaran, Rush, Crowther, &c. and drew up the extensive tables which are here condensed and put into a more easy form for reference. The population census they refer to was taken in 1825.]

### Tables.

#### I. *Number of Lunatics in Norway, and Nature of the Affection.*

| Disease. | Number. | | Curable. | | Incurable. | |
|---|---|---|---|---|---|---|
| | Male. | Female. | Male. | Female. | Male. | Female. |
| Mania .......... . | 270 | 242 | 84 | 74 | 186 | 168 |
| Melancholia .......... | 198 | 178 | 107 | 102 | 91 | 76 |
| Dementia .......... | 168 | 173 | 60 | 60 | 108 | 113 |
| Idiocy .......... | 369 | 311 | | | 369 | 311 |
| | 1005 | 904 | 251 | 236 | 754 | 668 |
| | 1909 | | 487 | | 1422 | |

#### II. *Proportion of Lunatics to the Population.*

| | Male. | Female. | Both. |
|---|---|---|---|
| Population .......... | 511033 | 540285 | 1051318 |
| Proportion of Lunatics ... | 1 in 508½ | 1 in 597½ | 1 in 550½ |

* Beretning, Betænkning og Indstilling fra en til at undersöge de Sindssvages Kaar i Norge, og gjöre Forslag til deres Forbedring i Aaret 1825, naadigst nedsat Kongelig Commission. Ifolge Kongelig Resolution udgivet af Frederick Holst, m.d. Christiania, 1828.

A Report from a Commission appointed by the King in 1825, to examine the Condition of Lunatics in Norway, and propose a Plan for its Improvement. Published, by Order of the King, by Frederick Holst, m.d., &c. Christiania, 1828.

### III. *Ages of Lunatics.*

| Ages. | Mania. | | Melancholia. | | Dementia. | | Idiocy. | |
|---|---|---|---|---|---|---|---|---|
| Years. | Male. | Fem. | Male. | Fem. | Male. | Fem. | Male. | Fem. |
| 0 — 5 | 1 | ... | .. | ... | ... | .. | 4 | 1 |
| 5 — 10 | 7 | 5 | ... | ... | 6 | 1 | 32 | 19 |
| 10 — 15 | 15 | 10 | ... | ... | 2 | 3 | 44 | 33 |
| 15 — 20 | 13 | 12 | 2 | 3 | 12 | 8 | 50 | 46 |
| 20 — 25 | 19 | 18 | 6 | 6 | 14 | 13 | 62 | 46 |
| 25 — 30 | 22 | 18 | 13 | 18 | 20 | 12 | 42 | 40 |
| 30 — 40 | 68 | 53 | 45 | 35 | 45 | 32 | 56 | 53 |
| 40 — 50 | 45 | 50 | 35 | 35 | 33 | 38 | 37 | 32 |
| 50 — 60 | 37 | 34 | 51 | 37 | 21 | 25 | 19 | 19 |
| Above 60 | 37 | 39 | 44 | 42 | 14 | 41 | 22 | 18 |
| Not mentioned | 6 | 3 | 2 | 2 | 1 | ... | 1 | 4 |
| Total ......... | 270 | 242 | 198 | 178 | 168 | 173 | 369 | 311 |
| | 512 | | 376 | | 341 | | 680 | |

### IV. *Number of Epileptics among the Lunatics.*

| | Mania. | | Melancholia. | | Dementia. | | Idiocy. | |
|---|---|---|---|---|---|---|---|---|
| | Male. | Fem. | Male. | Fem. | Male. | Fem. | Male. | Fem. |
| Epileptic ............... | 53 | 42 | 12 | 8 | 22 | 20 | 45 | 43 |

### V. *Duration of the Disease.*

| Duration. | Mania. | | Melancholia. | | Dementia. | | Idiocy. |
|---|---|---|---|---|---|---|---|
| Years. | Male. | Fem. | Male. | Fem. | Male. | Fem. | |
| 0 — 1 | 13 | 5 | 16 | 17 | 9 | 9 | Idiocy being assumed to be congenital, its duration is to be found in the Table of Ages of Lunatics. |
| 1 — 3 | 17 | 15 | 22 | 24 | 16 | 8 | |
| 3 — 5 | 21 | 20 | 25 | 23 | 16 | 15 | |
| 5 — 10 | 42 | 37 | 40 | 26 | 27 | 34 | |
| 10 — 15 | 36 | 36 | 26 | 32 | 22 | 23 | |
| 15 — 20 | 26 | 23 | 18 | 10 | 22 | 16 | |
| 20 — 30 | 42 | 31 | 26 | 20 | 27 | 27 | |
| 30 — 40 | 23 | 23 | 9 | 9 | 12 | 14 | |
| 40 — 50 | 10 | 8 | 3 | 2 | 2 | 5 | |
| 50 — 60 | 6 | 6 | 1 | 2 | 2 | 3 | |
| Above 60 | 1 | 1 | 1 | 1 | ... | 1 | |
| Not mentioned | 33 | 37 | 11 | 12 | 13 | 18 | |
| Total ...... | 270 | 242 | 198 | 178 | 168 | 173 | |
| | 512 | | 376 | | 341 | | |

VI. *Causes.*

| Physical Causes. | Mania. | | Melancholia. | | Dementia. | |
|---|---|---|---|---|---|---|
| | Male. | Fem. | Male. | Fem. | Male. | Fem. |
| Hereditary Teudency | 12 | 10 | 8 | 6 | 6 | 5 |
| Childbirth | ... | 6 | ... | 3 | ... | 7 |
| Epilepsy | ... | 1 | ... | ... | ... | ... |
| Apoplexy | 1 | ... | 1 | ... | 4 | 3 |
| Fever | 1 | ... | 5 | 2 | 12 | 5 |
| Blows on the Head | 2 | 1 | 2 | 2 | 7 | 2 |
| Water on the Brain | ... | ... | ... | ... | 1 | ... |
| Tumours in the Brain* | ... | ... | ... | ... | 1 | ... |
| Catching Cold | 2 | ... | 3 | ... | 1 | 2 |
| Rickets | ... | 1 | ... | ... | ... | ... |
| Weakness of Nerves | 1 | 1 | 3 | 5 | 2 | ... |
| Hysteria | ... | ... | ... | 5 | ... | 1 |
| Debauchery | 2 | 1 | 1 | ... | 2 | 1 |
| Drink | 10 | 1 | 10 | ... | 4 | ... |
| Masturbatio | 2 | ... | 1 | ... | 7 | ... |
| Total from Physical Causes | 33 | 22 | 34 | 23 | 47 | 26 |

VII. *Causes.*

| Moral Causes. | Mania. | | Melancholia. | | Dementia. | |
|---|---|---|---|---|---|---|
| | Male. | Fem. | Male. | Fem. | Male. | Fem. |
| Neglected Education | ... | ... | ... | ... | ... | 1 |
| Harsh Treatment | 2 | 1 | 2 | 2 | 3 | 1 |
| Disappointed Hopes | 4 | 2 | 1 | ... | ... | 1 |
| Hopeless Love | 13 | 34 | 11 | 29 | 5 | 21 |
| Unhappy Marriage | 1 | 5 | 1 | 2 | ... | 1 |
| Jealousy | 2 | 2 | 1 | 3 | 1 | 2 |
| Care | 6 | 7 | 33 | 18 | 7 | 15 |
| Fright | 18 | 13 | 8 | 5 | 10 | 10 |
| Fanaticism | 6 | 3 | 6 | 12 | 1 | ... |
| Scruples of Conscience | 2 | 2 | 9 | 3 | 1 | 1 |
| Injured Self-Love | 2 | ... | 3 | 2 | 2 | ... |
| Excess of Study | ... | ... | 4 | 2 | 1 | ... |
| Passionateness† | 1 | 4 | 2 | 6 | 2 | 3 |
| Total from Moral Causes | 57 | 73 | 81 | 84 | 33 | 56 |
| Total from Physical Causes | 33 | 22 | 34 | 23 | 47 | 26 |
| Causes not assigned | 180 | 147 | 83 | 71 | 88 | 91 |
| Total | 270 | 242 | 198 | 178 | 168 | 173 |

* We are not sure that we have here rendered the original by the proper word. The Danish *Byld,* no doubt the original of our *Boil,* may mean either a tumour or an abscess. It is sufficient to know that it was a local cause discoverable externally.

† We have preferred this term to *anger,* as better expressing a proneness to anger or passion, which the original term implies.

*Hospitals at Paris.*

[The following is the most recent account we have met with of the hospital establishments in the French capital; and, as we consider it authentic, we present it to our readers, as an interesting illustration at once of the very extensive provision made for the relief of distress, and of the vast opportunities for seeing disease afforded to the profession in Paris.]

*Hôtel Dieu;* 1000 beds.—*L'Hôpital de la Pitié;* 600 beds.—*L'Hôpital de la Charité;* 300 beds.—*L'Hôpital Saint-Antoine;* 250 beds.—*L'Hôpital Cochin;* 200 beds.—*L'Hôpital Beaujon;* 180 beds.—*L'Hôpital Necker;* 140 beds.

All kinds of patients are admitted into these hospitals, except children, insane, incurable, lying-in-women, and venereal or chronic cases. At L'Hôpital Necker there is a separate department for the treatment of stone by lithotrity.

*L'Hôpital des Enfans.* Hospital for children; 550 beds for children of both sexes, from two to fifteen years of age.

*L'Hôpital St. Louis;* 700 beds. Diseases of the skin, ulcers, scrofula, &c. Gratuitous advice daily; vapour, sulphur, and other baths.

*L'Hôpital des Vénériens.* Venereal hospital; 650 beds.

*Maison Royale de Santé;* 175 beds. This institution is for the diseased, or wounded, who are not able to have attendance in their own houses, but who pay from three to six francs daily.

*Maison d'Accouchement.—Maternité.* Lying-in-hospital; 350 beds. Besides these twelve hospitals, there are ten institutions or asylums (*Hospices*).

*Hospices des Enfans-Trouvés;* 258 beds. Infants deserted by their parents are received here, and suckled.

*Two Hospices for the Old.—La Salpêtrière,* which contains room for 5,100 women; and *Bicêtre,* for 3,200 men.

*Two Hospices for the Incurable.* Women and children, 525 beds. Men, 455 beds.

*Hospice Larochefoucauld;* 200 beds. Asylum for the servants of these institutions, the indigent of both sexes, the aged or infirm, pensioners.

*Hospice de Orphelins.* Orphan asylum; 750 beds. Half for girls, and half for boys. For children abandoned by their parents, and kept until their majority.

*Institution de Sainte-Périne;* 175 beds. Aged or infirm of both sexes, who pay a certain sum.

*Hospices des Ménages;* 670 beds. Indigent married people. Women at least sixty years old, and men seventy. Widows and widowers at sixty.

*Hospice St. Michel à St. Maudé;* 12 beds for old men of seventy years of age. Founded by M. Boulard, a merchant.

There are therefore, in Paris, 16,549 beds for patients.

Besides these hospitals and asylums, there are a great number of other benevolent institutions.—*La Lancette Française,* No. xciii. 6 *Août,* 1835.

---

*On the Means of increasing the Number of Leeches in France.* By M. GUIBOURT.

[A REPRESENTATION having been made to the French government on the subject of preserving and augmenting the number of leeches in France, the opinion of the Academy of Medicine was requested by the minister, and a commission was appointed to make a report on the subject. This report, drawn up by M. Guibourt, was read at the sitting of the Academy on the 22d September last; and as it contains some very interesting details on matters of no slight importance to all who practise medicine, we shall extract a considerable portion of it. The subject becomes more interesting when we reflect that we, as well as the French, derive our supply of leeches from foreign countries.]

Twenty-five years ago more than enough leeches were produced in France to supply the whole demand, at the small price of 15 to 60 francs a thousand. But soon after this, the consumption became so much greater that they were obliged to be obtained from Belgium, Spain, Italy, Bohemia, and even Africa. At present few are procured in France, although the price is 150 to 250 francs a thousand: Brittany and Sologne alone furnish a few for commerce; in other places the quantity does not supply the local wants. Spain and Bohemia are also exhausted: Tuscany affords only an inferior sort, and the vast marshes of Hungary are beginning to be depopulated, so that they are now obtained at the establishment of Vertus near Paris from the frontiers of Russia and Turkey. They are brought from these countries to Palota near Pest, where they are kept in reservoirs belonging to the same establishment, and sent on to Paris as required. At Palota they are packed up in bags, each of which contains from 50 to 70 lb. weight, and these bags are arranged side by side in hammocks, suspended in a light covered cart, like that used by upholsterers, which travels post to Paris in twelve or fourteen days. They never arrive there without delays, for in hot and stormy weather it is necessary to refresh them with water, sometimes twice, but at least once daily. For this purpose there are at Kell large troughs, in which smaller ones are placed, and both are filled with water. The sacs of leeches are emptied into the little troughs, from which the animals escape into the greater ones, and all those which remain at the bottom of the water are rejected as unable to bear the journey. On their arrival at Vertus establishment, they are placed in large reservoirs of running water, of which the banks are planted with reeds. They remain there generally a month, but when the demand is great they have only five or six days' rest, and as they are exhausted with the journey, they are complained of as bad leeches. Young leeches, supposed to have been born on the establishment, are seldom seen, and they are at least eight years attaining their full size: this may not be the natural period of their growth, for the adult leeches grow thin instead of increasing in size in captivity. M. Durand, apothecary at Montereau, has however seen leeches propagate in reservoirs where he keeps them, and he has sent to the academy leeches of from one to two years old. These, however, are not one quarter the full size, and they would therefore take eight years to attain it. This would make them more expensive than if imported. In order to prevent the failure of the supply of leeches, M. Guibourt proposes that those which have been used in the Paris hospitals, (500,000 annually,) should be sent into marshy and uninhabited districts of France to multiply: pits should be dug in the numerous little islands in the rivers for their reception, so that, when winter came, and these islands were overflown, the leeches would be dispersed, and when at full liberty would become healthy, and increase in number; and in a few years there would be less necessity to seek them in foreign countries. An experiment was for some time made with the old leeches of the Hôtel Dieu, which were placed in a pond supplied by a stream, and in five or six months it was found that they could be reapplied. As 70,000 francs are spent annually in the French hospitals for leeches, the question of supply is important.

In a discussion which followed the reading of this report of the Academy, M. Bouillaud stated that he formerly used from 8 to 10,000 annually, but that now he had substituted cupping, and only uses 500. He cups for erysipelas, and on joints attacked with acute inflammation. M. Lisfranc thought leeches were preferable, not only in particular situations where cupping could not be performed, but from their producing a revulsive irritation in the skin, which is attended with excellent effects.

*Gazette Médicale de Paris*, No. 40, 3 Oct. 1835.

